# Autistic Children Have a Voice and Want to Use It: Acceptability of Dyadic, Telehealth, Occupation-Based Coaching

**DOI:** 10.63144/ijt.2026.6761

**Published:** 2026-06-01

**Authors:** Melanie Morriss Tkach, Winnie Dunn, Nathaniel Dare, Timothy J. Wolf

**Affiliations:** Department of Occupational Therapy, College of Health Sciences, University of Missouri, Columbia, MO, USA

**Keywords:** Autism Spectrum Disorder, Occupation-based coaching, Occupational therapy, Telehealth

## Abstract

Autistic children may need additional support to manage daily activities compared with their peers. Telehealth occupation-based coaching (OBC) may help them participate in daily activities and develop related skills. Although telehealth OBC is feasible and acceptable to caregivers, its acceptability to children remains unknown. This phenomenological study explored the lived experiences of seven children who completed dyadic, telehealth OBC with their caregivers. We conducted semi-structured interviews and thematic analysis to understand their perspectives on intervention acceptability. Three themes emerged: (1) *I want to be included and heard in coaching calls*, (2) *I can learn to participate in coaching*, and (3) *I like coaching because I met my goals*. Findings suggest that dyadic, telehealth OBC is acceptable to children because it supports their inclusion, fosters self-efficacy in coaching, and contributes to perceived improvements in daily activities. Findings also provide preliminary evidence that this approach can promote child engagement and self-determination.

Children and youth typically assume responsibility for daily activities over a 5 – 15-year period, and no formalized process exists to support the transition (Kao et al., 2021). In many families, children gradually take on responsibility by participating in everyday activities alongside caregivers, who provide direct teaching, modeling, oversight, and feedback (Kao et al., 2021; Munsell et al., 2023). Autistic children and youth may require more time to take on responsibilities because they typically participate less across contexts and rely on caregiver support for longer periods of time than non-autistic peers (Davy et al., 2022; Egilson et al., 2017, 2018; Krieger et al., 2018; Krieger et al., 2024; Lamash et al., 2020; Ratcliff et al., 2018; Simpson et al., 2019). As a result of lower participation rates and prolonged caregiver involvement, autistic children may have fewer opportunities to develop skills that lay a foundation for self-determination and a successful transition to adulthood (Palmer et al., 2017; Ryan & Deci, 2017; Wehmeyer, 2014).

Foundational skills for self-determination, such as setting goals, making choices and decisions, and problem solving, emerge in childhood through participation in meaningful activities. These skills enable children and youth to exercise self-determination, or take action to reach personal goals and lead a meaningful and fulfilling life, in their natural contexts (KU Center on Disabilities, 2024; Wehmeyer, 2014). Children develop foundational self-determination skills and form beliefs about themselves and their ability to influence the environments around them as they participate in meaningful activities (Palmer et al., 2017). Because children are still largely dependent on trusted adults, the social environment plays a pivotal role in shaping emerging self-determination skills. When caregivers offer meaningful choices, structure the environment to promote competence, and invest time and attention, children are more likely to develop these skills (Palmer et al., 2017; Ryan & Deci, 2017; Wehmeyer, 2014). Rehabilitation interventions must intentionally create opportunities for activity participation and scaffold supportive environments to foster self-determination in autistic children and adolescents (Ryan & Deci, 2017; Wehmeyer, 2014; Wehmeyer & Shogren, 2017).

Occupation-based coaching (OBC) can support the development of foundational self-determination skills in childhood. OBC uses a collaborative problem-solving approach to address activity-based goals and situates the intervention within families’ natural routines and contexts (Dunn et al., 2018). During OBC, families have opportunities to make meaningful choices about goals, share their lived experiences, and co-create and implement strategies that foster child success. OBC with caregivers of young autistic children improves goal achievement, occupational performance, and caregiver efficacy (Little et al., 2018; Little et al., 2023). However, aside from our previous study, there is limited research on OBC with autistic children or guidelines for engaging them in the coaching process.

Engagement is a proposed mechanism of change for OBC and may serve as a critical pathway through which OBC fosters self-determination and other positive outcomes. Engagement involves an emotional connection with the provider, enthusiasm for the intervention, beliefs that intervention is necessary and can be effective, and active participation in therapeutic activities (King et al., 2020; King et al., 2014). Children demonstrate engagement through observable behaviors such as taking ownership of therapeutic activities, sustaining attention, and relating to the provider (D’Arrigo et al., 2020a). However, engagement is dynamic and may fluctuate within and across sessions due to multiple interrelated factors including daily circumstances, caregiver involvement, fatigue, or developmental changes.

When serving children and families, providers foster child engagement and foundational skills for self-determination through relational strategies such as building rapport, responding flexibly to child and family needs, validating emotions, setting expectations, offering meaningful choices, and providing just right challenges (D’Arrigo et al., 2020b). These strategies create structured opportunities for children to practice goal setting, decision making, and problem solving with support and performance feedback. Although much of the existing child engagement literature pertains to in-person therapy, we extended these principles to telehealth OBC for the current study; in doing so, we aimed to create authentic therapeutic environments and interactions that empower children and families to achieve their goals.

Previously, we tested the feasibility of telehealth OBC for autistic children and their caregivers (Tkach et al., 2025). Telehealth OBC was feasible, acceptable to caregivers, and yielded promising effects for both child and family outcomes. Additionally, twelve of 13 children actively participated in telehealth OBC calls with six sharing insights and generating strategies, three sharing insights only, and three primarily connecting with the provider (Tkach et al., 2025). Although these results offer preliminary evidence for the feasibility of dyadic, telehealth OBC and behavioral indicators of child engagement, they do not fully capture intervention acceptability from the child perspective nor the affective and cognitive elements of child engagement.

Therefore, we explored the lived experiences of autistic children who participated in telehealth OBC with their caregivers through qualitative interviews. Qualitative data can capture children’s perceptions of the acceptability of dyadic, telehealth OBC by inquiring about their cognitive and affective responses to the intervention (Patton, 2015; Sekhon et al., 2017). Understanding child perspectives about intervention acceptability and engagement in dyadic, telehealth OBC can help us refine the intervention and optimize child engagement to promote positive intervention outcomes, including self-determination.

## Methodology

### Design

We used a phenomenological approach to explore children’s lived experiences of dyadic, telehealth OBC. We conducted in-depth semi-structured interviews with a subset of children and caregivers who completed telehealth OBC as part of a prior feasibility study (Tkach et al., 2025). The prior study examined quantitative feasibility metrics and caregiver acceptability, whereas the present study builds on those findings through a qualitative exploration of child experiences and acceptability. The study was approved by the University of Missouri’s institutional review board. Caregivers provided electronic informed consent, and children provided verbal assent. We followed the COnsolidated criteria for REporting Qualitative research (COREQ) 32-item checklist to provide a comprehensive report (Tong et al., 2007).

### Participants

We used purposive sampling to enroll children and caregivers who completed the prior feasibility study (see Tkach et al., 2025 for detailed inclusion/exclusion criteria). We invited all families who completed the intervention to participate in an interview and conducted six interviews with six families comprised of seven caregivers and seven children. Three families were lost to follow-up, and two families declined participation due to concerns about recall bias.

Children who enrolled in the study were 7 – 10 years old (*M* = 8.71 years; *SD* = 1.38 years). All children were White and Non-Hispanic. Most children (n = 5) and caregivers (n = 6) completed one, 60-minute telehealth OBC session per week for eight weeks. One caregiver who had two children enrolled in the study discontinued telehealth OBC after seven sessions because her children met all established goals and had no additional concerns. See Table 1 for participant characteristics.

### Data Collection

Two doctoral candidates, one male (ND) and one female, conducted semi-structured interviews. Interviewers were not previously known to the children or caregivers who participated. Both interviewers were registered and licensed occupational therapists with prior experience interviewing children and families in clinical practice, and they received training on the semi-structured interview guide before data collection commenced. Participants knew that interviewers were interested in learning about their experiences with dyadic, telehealth OBC.

Interviews were conducted using Protected Zoom, a secure videoconferencing service with end-to-end encryption in compliance with the Health Insurance Portability and Accountability Act of 1996 (HIPAA; Pub. L. 104–191). All interviews were video- and audio-recorded for transcription and data analysis. Interviews lasted between 20 and 57 minutes, depending on the number of caregivers and/or children on the call, the child’s age, and the child’s language level.

Interviewers utilized a semi-structured interview guide with questions based on the Theoretical Framework of Acceptability and existing coaching studies conducted with caregivers of autistic children (Sekhon et al., 2017; Smith et al., 2023; Wallisch et al., 2019). See Table 2 for a list of interview questions.

Interviewers integrated strategies to support effective and ethical inclusion of autistic people in research as recommended by the Academic Autism Spectrum Partnership in Research and Education (AASPIRE). They offered children a break every 30 minutes or after half of the interview questions had been answered (AASPIRE, 2020). They also used consistent phrases for acknowledging child contributions, probing for deeper responses, and moving the conversation forward (AASPIRE, 2020; Nicolaidis, 2020).

### Data Analysis

The analytic team included two occupational therapy doctorate students who served as primary coders and two licensed occupational therapists, one a doctoral candidate (ND) and one with a research doctorate (MT), who facilitated decision-making during full team meetings. We transcribed interviews verbatim and cleaned transcripts according to the Automatic Alignment and Analysis of Linguistic Change – Transcription Guidelines (Linguistic Data Consortium, 2003). We analyzed the interview data using Braun and Clarke’s guidelines for thematic analysis (Braun & Clarke, 2006). The two primary coders independently reviewed two transcripts to familiarize themselves with the data and generated initial codes for child responses and observable behaviors. Then, the full team met to resolve discrepancies, establish consistent terminology, and develop a formal codebook. The two primary coders recoded transcripts according to the formal codebook and achieved adequate intercoder reliability (κ = 0.85). After reaching intercoder reliability, they independently coded the remaining transcripts and generated preliminary themes. The full team met to review and refine preliminary themes, cross-checking each theme with coded data. We used visual mapping and iterative discussions to finalize theme names, definitions, and examples for the written report (Braun & Clarke, 2006).

### Trustworthiness

We reflected on personal backgrounds, perceptions, and interests to consider how these factors might influence the qualitative research process (Krefting, 1991). All research team members had occupational therapy training, were interested in coaching interventions for children and families, and valued including children’s perspectives in therapy. To address potential influences on data interpretation, we engaged in ongoing reflexive dialogue and maintained field notes to document observations, insights, and emotional responses during coding sessions and team discussions (Krefting, 1991). We collected and analyzed interview data and reached mutual confirmation when interpreting data to minimize bias from a single researcher (Krefting, 1991). Additionally, a peer examiner who is a senior scholar with OBC expertise met with the research team to clarify the research question, formalize the coding process and codebook, and refine emerging themes. Throughout data analysis, the peer examiner asked probing questions to challenge team assumptions (Krefting, 1991).

## Results

Children shared their perceptions of dyadic, telehealth OBC through their words and behaviors. Three overarching themes emerged from the data, shedding light on intervention acceptability from the child’s point of view: (1) I want to be included and heard in coaching calls, (2) I can learn to participate in coaching, and (3) I like coaching because I met my goals.

### I want to be included and heard in coaching calls

During interviews, children communicated a desire to be included and heard through their words and actions, though depth of responses and the levels of caregiver support needed varied by age. Younger children often provided brief, one-word responses and relied on caregivers to help them recall the coaching experience or maintain focus. In contrast, older children elaborated on their responses with examples and explanations, and they engaged in more reciprocal dialogue with caregivers when answering questions. See Table 3 for examples of child responses and corresponding caregiver support levels by age.

Children participated in coaching calls by clarifying questions, refining their responses, and directing aspects of the conversation. They acknowledged when they lost track of a question, stating, “I also kind of forgot what the question was” (Participant 11) and requested reminders to refocus, asking, “What’s the question again?” (Participant 19). They also requested that the provider or caregiver(s) define unfamiliar words or phrases to support their understanding, asking, “What does that mean?” (Participant 34) and “What’s experience?” (Participant 23).

Children refined their responses to ensure accurate interpretation. For example, when a caregiver described one child’s prior “tantrums,” the child responded:

“Tantrum is the wrong word for it. The word that I think would be most similar to it would probably be like…I never threw things because I didn’t…” (Participant 11).

Through discussion, the family ultimately agreed upon the term “outbursts,” illustrating how the child used his voice to shape the language used to describe his experiences.

Children also directed the flow of conversation. To connect with the provider, some children asked questions, such as, “Where are you [interviewer]?” (Participant 35) or “Have you heard of [Raising] Cane’s?” (Participant 34). They also shared personal interests, including a favorite book about mystical creatures. To guide the conversation and make choices about its structure, some children asked, “Can I start with my answer?” (Participant 34) or prompted caregivers to speak first, stating, “Share what you guys think maybe…” (Participant 11).

Alongside participation, children demonstrated their desire to be included and heard during coaching calls by expressing and managing their sensory, emotional, and behavioral needs to maintain engagement. Younger children often redirected attention to their immediate needs by requesting drinks, snacks, or tablets to play games, whereas older children actively implemented self-regulation strategies. For example, one child said, “Also, can we switch spots because my legs are getting a little bit sore in this area, and I just want to be able to stand up…” (Participant 11). Another child explained his need to alternate between responding and interacting with a pet for comfort, saying, “I’m gonna go back to playing with the kittens now” (Participant 19).

Additionally, older children minimized distractions in the home environment to maintain focus during coaching conversations. One child demonstrated this during the interview, saying, “So sorry I'm gonna turn that [fan] down a little bit,” and later added, “If I can, go let our dog into my room…” when the pet was scratching at the door (Participant 11).

Finally, children demonstrated their desire to be included and heard in coaching conversations by reflecting on aspects of the environment that facilitated their engagement during coaching calls. One child noted, “...it’s our home, and that’s still pretty comfortable” (Participant 19), suggesting that the familiarity and comfort of home may support children’s ability to regulate, remain engaged, and communicate their thoughts. Another child explained, “And I think it was also easier to do it with people, you know, in the room. And at a place…where you can cool down and stop easier” (Participant 11), highlighting the importance of supportive caregivers in fostering a sense of inclusion. However, personal items within the home environment, such as toys, electronics, and pets, occasionally distracted children and interfered with their engagement in the intervention.

### I can learn to participate in coaching

Children exhibited emerging self-efficacy with coaching skills by reflecting on activity performance and exploring new ways to complete activities. Children frequently shared the activity-based goals that they worked on during coaching calls such as putting laundry in a basket (Participant 19), sleeping in their own bed (Participant 23), brushing teeth (Participant 30), and tying shoes (Participants 34 & 35). They also evaluated how well they performed goal activities. For example, when asked about her goal to manage emotions during transitions from electronics to the nighttime routine, one child said, “Um, kind of better ‘cause I still throw fits,” demonstrating awareness of her performance and areas for continued growth (Participant 23). Another child reflected on his goal, saying, “It was pretty easy. All you have, all you have to do is just put the clothes in the basket,” showing confidence in his ability to complete the activity (Participant 19).

Children also demonstrated that they were learning how to participate in coaching by exploring new ways to complete activities with support. Younger children primarily listened to the conversation and communicated agreement or disagreement with the plan, whereas older children described how they modify activities or the environment to improve their performance. For example, one older child explained a strategy that helped him put his dirty laundry in a basket, “What I think is like if it’s…a game and it’s fun then I kind of want to do it again because it’s fun” (Participant 19). Similarly, another child shared that creating a story to say during the shoe-tying sequence was helpful, noting, “Um…the story of how to tie your shoes is like…funny” and continued to brainstorm additional strategies during the interview, suggesting, “Maybe a song for it [shoe tying]?” (Participant 34). One older child and his caregivers identified the flavor of his medication as a barrier, so they switched from liquid to capsules. They applied the same insight to the flavor of his toothpaste and noticed improvements in his willingness to brush consistently over time, as illustrated in the following exchange:

Participant 11: “And then we have to stop by to get some sort of soap for our bathroom. And I'll be like, Hey, I need toothpaste. Can I maybe get some this time?”

Caregiver: Yeah, so you said that so nicely, whereas before you just wouldn't brush your teeth [laughs].

Although children demonstrated emerging coaching skills, many described difficulties sustaining attention during coaching calls. One older child explained, “And like it’s just hard…for someone who has ADHD and autism, it was just hard for me to focus on one thing” (Participant 11). Another child noted how basic physiological needs affected his participation, stating, “The only times it was hard was when I wanted to eat or when I wanted to go to bed” (Participant 19). Despite these challenges, younger children communicated their needs to caregivers to obtain assistance, while older children implemented self-regulation strategies to recenter and rejoin the conversation, as described above.

### I like coaching because I met my goals

Children reported improvements in their activity performance and underlying performance skills through coaching and associated these gains with positive perceptions of the intervention. Across ages, children shared that they met activity-based goals in statements like, “I’m brushing my teeth” (Participant 19) or “I can tie shoes and help people” (Participant 34). Another child added that coaching helped him prioritize activities, explaining, “And I think it was just… small things about like remembering to do like just regular things that I need to do in…a day or something and then like doing what I want to do after it” (Participant 11).

Older children also described improvements in skills that support daily activities. When asked what he found helpful about coaching, one child explained, “Oh…what I can do to fix the problems…like the bugs issue” (Participant 31), indicating that coaching helped him identify strategies to navigate his fear of bugs in daily life. Another child shared, “…it’s [OBC] helped me communicate with them [caregivers] better…”, a sentiment echoed by his caregivers (Participant 11). The same child added, “I just noticed that it [OBC] like helped me not…get upset as quickly,” and “…I did not like to do things before that were out of my comfort zone. But I’m a lot better at that now than I was before we took it [OBC]” (Participant 11).

Children conveyed that perceived improvements shaped how they viewed the intervention. One child noted, “It [OBC] helped me…made me be able to do, accomplish one of my goals. Maybe not for all the younger kids, but…I like it” (Participant 19). When asked what excited him about the coaching calls, another child shared, “Well, at first, I wasn't really wanting to do it, because...I didn't usually take my medicine as often because, as I said, that's one of the things that it helped me do... I think that it was just good...I just like started to actually notice improvement after from it” (Participant 11). He revisited this idea later in the interview saying, “…It just helped me to know that I was both helping…not just myself get better with it [Autism] but also other people probably who might also take this thing [OBC]...” (Participant 11). In contrast, younger children preferred external rewards rather than perceived improvements to stay engaged. As one child asked, “How are we going to know we did a good job without a reward?” (Participant 34).

## Discussion

The purpose of this study was to explore lived experiences of children who completed dyadic, telehealth OBC with their caregivers. Child perspectives indicated alignment between the intervention and their desire for inclusion, their emerging sense of self-efficacy in the coaching process, and their perceptions of coaching effectiveness. Collectively, these findings deepen our understanding of intervention acceptability according to children and provide preliminary evidence of their engagement and foundational self-determination skills in the context of dyadic, telehealth OBC.

### I want to be included and heard in coaching calls

Children communicated a desire to be included in coaching calls by actively joining the conversation, managing sensory, emotional, and behavioral needs to stay engaged, and identifying supports, such as the comfort of the home environment, that fostered inclusion. Their consistent efforts to engage in dyadic, telehealth OBC suggest that the intervention aligned with their value of inclusivity, thereby demonstrating its ethicality (Sekhon et al., 2017). This finding may be explained by the collaborative nature of OBC, which positions children and families as experts in their lived experiences and empowers them to create, implement, and evaluate plans that support goal progress and achievement (Dunn et al., 2018).

This theme extends prior research on child involvement in pediatric therapy and individualized education plan (IEP) meetings. Existing studies show that including children’s voices in both contexts is feasible and beneficial, as children can create meaningful goals, learn participation strategies, and develop self-determination skills through active participation (Chandroo et al., 2018; Curtis et al., 2022; Sanderson & Goldman, 2020). Our findings add to this literature by revealing that children not only have the ability to participate but express a desire to be involved throughout the coaching process as well. Embedding children’s voices throughout therapy and in IEP meetings may, therefore, enhance engagement and foster self-determination as they prepare for adulthood (Chandroo et al., 2018; Curtis et al., 2022; O’Connor et al., 2021).

Relatedness, a key element of self-determination theory, may also be central to children’s experience of inclusion. Prior research demonstrates that collaborative provider-caregiver relationships help caregivers feel supported during coaching interventions (Chien et al., 2020; Wallisch et al., 2019). Therefore, collaborative relationships among providers, children, and caregivers are likely to promote engagement and a sense of inclusion during coaching calls. Relational strategies commonly used in pediatric therapy and interventions to increase IEP participation, along with autonomy-supportive parenting practices, may cultivate a safe and responsive social environment that fosters the development of self-determination skills (Curtis et al., 2022; D’Arrigo et al., 2020b; Palmer et al., 2017; Ryan & Deci, 2017; Wehmeyer, 2014).

### I can learn to participate in coaching

Children demonstrated emerging sense of self-efficacy with the coaching process, even when internal states posed challenges. Many reported growing confidence in their ability to reflect, problem-solve, and implement strategies with caregiver support (Sekhon et al., 2017). Even when attention waned or they felt hungry or tired, children communicated their needs and used self-regulation strategies to remain engaged. These behaviors suggest that children viewed the intervention as worth the effort, especially older participants (Sekhon et al., 2017).

This finding extends prior reports of perceived gains in caregiver self-efficacy after coaching to children themselves (Angelin et al., 2021; Graham et al., 2014; Wallisch et al., 2019). Although children in our sample gained confidence with key coaching skills, caregiver support appeared to play an important role in facilitating these gains and varied by age, cognitive and language abilities, and other internal states. Caregivers often supported children during coaching calls and follow-up interviews by restating or clarifying questions, helping them recall relevant events, answering first, and prompting elaboration. Providers and caregivers can further grade coaching demands to a child’s abilities and needs through strategies such as providing common coaching questions in advance, asking either-or questions to move the conversation forward, offering breaks, or providing alternate response formats to promote child success (Antoniadou et al., 2024; Curtis et al., 2022; D’Arrigo et al., 2020b; King et al., 2022; Nicolaidis et al., 2019). By doing so, we can create space for child engagement and development of self-determination skills in telehealth coaching.

### I like coaching because I met my goals

Finally, children indicated that perceived effectiveness played an important role in their affective attitudes toward the intervention (Sekhon et al., 2017). Our findings offer one of the first qualitative accounts of children’s direct perceptions of progress within telehealth coaching. Younger participants conveyed satisfaction and pride in their accomplishments through their body language and tone, whereas older participants explicitly identified progress as a primary reason they liked the intervention. These perceived gains are consistent with prior research that describes caregiver-reported improvements in children’s activity performance and participation after coaching (Chien et al., 2020; Graham et al., 2014). Moreover, this finding demonstrates that children were cognitively engaged in the intervention, and their emerging beliefs about coaching effectiveness contributed to positive affect towards the intervention (King et al., 2020; King et al., 2014; Sekhon et al., 2017).

### Limitations

This study included a small sample of White, non-Hispanic children aged 7–10 years, which limits transferability of findings to autistic children from more racially and ethnically diverse backgrounds, different age groups, those with minimal speaking abilities, and those with varying access to digital technology. Additionally, the sample included children with a range of ages, cognition, and communication abilities, which influenced the length, relevance, and depth of their responses. As a result, themes may be more reflective of the perspectives of older autistic children. Because we were unable to interview all children who completed the intervention, the views of those who declined participation or were lost to follow-up may differ from those presented here. Caregiver support facilitated child participation in coaching calls and follow-up interviews but may have influenced the perspectives children ultimately shared. Although we incorporated trustworthiness techniques to reduce potential bias, the research team was familiar with coaching interventions and strategies to include children in therapy, which may have influenced the interpretation of findings.

### Implications

Outcomes from this study inform the following next steps for providers and researchers:

- Dyadic, telehealth OBC appears acceptable to children, as evidenced by themes aligned with key intervention acceptability constructs (i.e., ethicality, self-efficacy, perceived effectiveness, affective attitude).
- Children want to be included in coaching calls and can learn to participate with tailored support to grade coaching demands and self-regulate internal states.
- Future research should examine how relational strategies and autonomy-supportive parenting practices influence child engagement and the development of self-determination skills in telehealth coaching.
- Future research should also broaden fidelity protocols to incorporate considerations for dyadic and family applications of telehealth OBC.

## Conclusion

We explored the lived experiences of children who completed dyadic, telehealth OBC to understand intervention acceptability from their perspectives. Children communicated that OBC was acceptable because it supported their inclusion in the therapy process, promoted self-efficacy in coaching activities, and resulted in perceived improvements in their daily lives. The collaborative nature of OBC, the comfort of participating from home, and support from both providers and caregivers fostered child engagement in the intervention and created opportunities for children to practice emerging self-determination skills. Future research should investigate how relational strategies and autonomy-supportive parenting practices impact engagement during coaching calls and expand fidelity protocols to include considerations for dyadic and family applications.

## Figures and Tables

**Table 1:** Child Demographics

Participant ID	Age (Years)	Gender	Comorbidities	Family Member(s) Present
11	10	M	ADHD	Mother and father
19	10	M	ADHD, LD	Mother
23	7	F	None	Mother
30	7	M	None	Mother
31	9	M	ADHD	Mother
34	8	F	None	Mother and brother (Participant 35)
35	10	M	None	Mother and sister (Participant 34)

*Note.* M = male; F = female; ADHD = attention deficit hyperactivity disorder; LD = learning disability.

**Table 2:** Semi-Structured Interview Questions for Children and Caregivers

Question #	Interview Question
1	What did you like most about the coaching calls on Zoom?
2	What was the most helpful part of the coaching calls on Zoom?
3	What kept your attention or got you excited to talk during the coaching calls on Zoom?
4	What, if anything, are you doing differently after the telehealth intervention?
5	How do you problem-solve challenges now?
6	What was the hardest part of the coaching calls on Zoom?
7	What could we do better in the coaching calls on Zoom so that they are more helpful?
8	What could we add to the coaching calls on Zoom so that they are more helpful?
9	What can we do differently to keep your family’s attention and get you excited to talk during the coaching calls on Zoom?
10	What would you like to share with other kids or parents about this experience?

**Table 3:** Examples of Child Responses and Caregiver Support by Age

Interview Question	Child Response	Caregiver Support
What did you like most about the coaching calls on Zoom?	“Like about like, uh, 8.5...out of 10.” (Participant 23, 7F)	Restated and clarified question, provided three or more contextual details, encouraged response
	“Is it [taking medicine] something that I’m doing better at? Yes. But it just helped me do that, and we communicate better and stuff.” (Participant 11, 10M)	Provided two contextual details, encouraged response
What was the most helpful part of the coaching calls on Zoom?	“Brushing to your teeth before school, even at nighttime.” (Participant 30, 7M)	Answered first, restated question, shared examples, restated question, encouraged response
	“That it was…remembering to do like the things that I have to do…like taking my medicine more often.” (Participant 11, 10M)	Answered first, clarified distinction between ‘liked” and “helpful’
What was the hardest part of the coaching calls on Zoom?	“To pay attention.” (Participant 23, 7F)	Answered first, shared examples, encouraged response
	“The only times it was hard was when I wanted to eat or when I wanted to go to bed.” (Participant 19, 10M)	Answered first, encouraged response

*Note.* F = female, M = male.
